# Dermoscopy of annular elastolytic giant cell granuloma^☆^

**DOI:** 10.1016/j.abd.2021.07.009

**Published:** 2022-09-22

**Authors:** Tiago Fernandes Gomes, José Carlos Cardoso, Victoria Guiote

**Affiliations:** a Dermatology Department, Centro Hospitalar de Leiria, Portugal; b Dermatology Department, Centro Hospitalar e Universitário de Coimbra, Portugal

**Keywords:** Alopecia, Dermoscopy, Granuloma, Hair disorders

## Abstract

Annular elastolytic giant cell granuloma is an uncommon granulomatous cutaneous disease that usually affects sun-exposed skin. Non-scarring alopecia is a possible presentation. Although histopathology is mandatory for the diagnosis, dermoscopy may help to narrow down the clinical differential diagnosis. The authors report a case of annular elastolytic giant cell granuloma in the scalp of a female adult patient, showing multiple yellowish/orange follicular dots in a diffuse erythemato-whitish background in the dermoscopy.

## Case report

**Figure 1:**
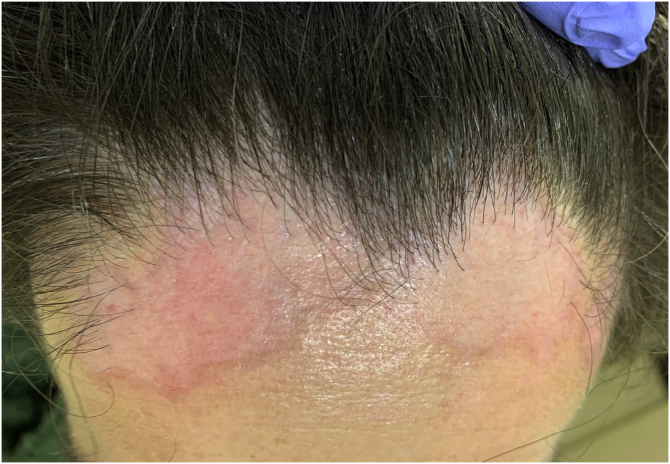
Pink patches with erythematous border at the frontal implantation hair line.

A 46-year-old female patient presented with a 2-month history of an enlarging frontal alopecic plaque. She denied any pain or pruritus. During the physical examination there were two confluent non-scaly pink alopecic plaques with an erythematous border, at the frontal implantation hair line, with 35 × 70 mm and 40 × 60 mm (Fig. 1). The overlying central skin was slightly atrophic. The remaining scalp had no decrease in hair density.

**Figure 2:**
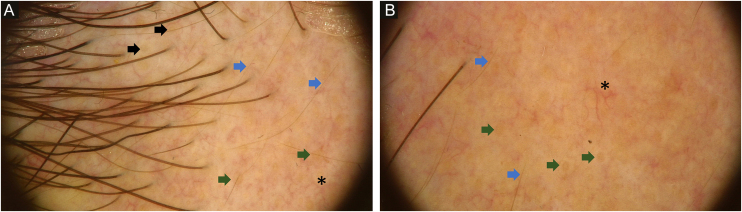
Diffuse erythemato-whitish background, with empty hair follicles (black arrow), yellow-orange dots (green arrow), pigmented thin hairs (blue arrows) and fine linear branching vessels (*).

**Figure 3:**
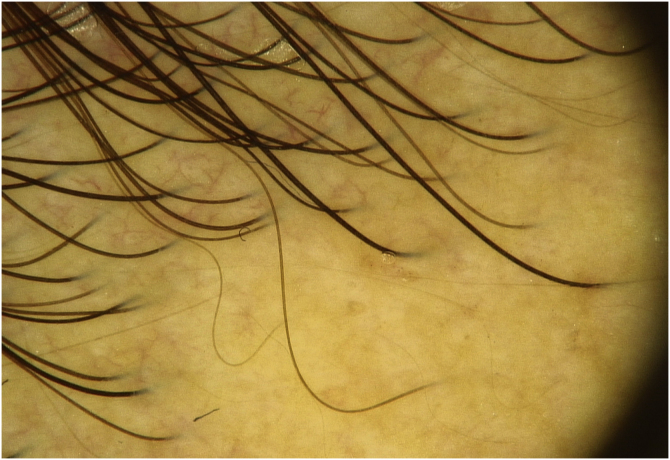
Dermoscopy of the unaffected skin of the scalp, at the frontotemporal hair line. There is no evidence of yellow dots.

**Figure 4:**
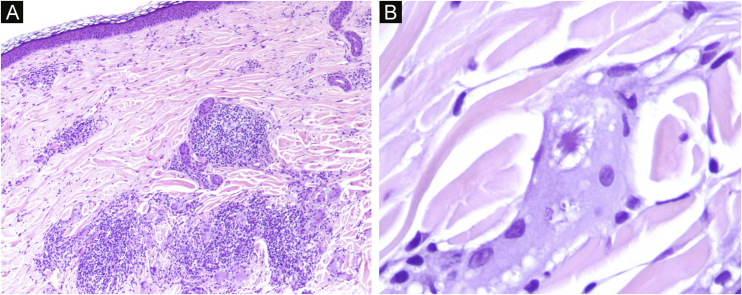
(a) Dermal lymphoplasmacytic infiltrate and granulomatous infiltrate with interstitial multinucleated giant cells. (b) Giant cell with intracitoplasmatic asteroid bodies. (a ‒ Hematoxylin & eosin, ×40; b ‒ Hematoxylin & eosin, ×400).

**Figure 5:**
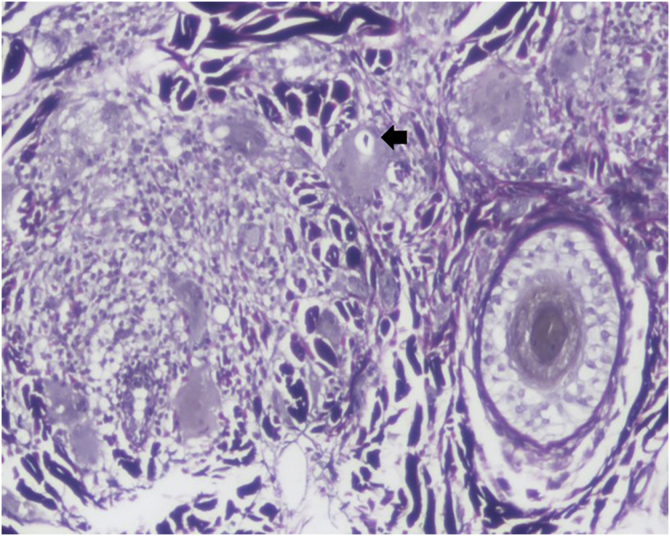
Elastic fibers in the cytoplasm of a multinucleated giant cell (black arrow). (Verhoeff-Van Gieson, ×100).

Dermoscopy revealed empty follicular ostia, with a diffuse erythemato-whitish background, with fine unfocused linear branching vessels. It was also evident multiple yellowish to orange follicular dots in the center of the alopecic patch, with some pigmented thin hairs (Fig. 2). When applying more pressure on the dermoscope, the erythematous background became more whitish, and the orange dots were more evident. No scale or hair shaft abnormalities were observed. Although the dermoscopy of the unaffected skin at the frontotemporal hair implantation line also revealed some pigmented thin hairs and linear branching vessels, no yellow dots or anysotrichosis were evident (Fig. 3). An incisional biopsy was performed. Histopathological examination revealed epithelial atrophy. In the mid and upper dermis, there was a granulomatous infiltrate with multinucleated giant cells, some of them with intracytoplasmic asteroid bodies, and there was also a sparse lymphoplasmacytic infiltrate (Fig. 4). No necrosis was evident. Verhoeff-Van Gieson stain showed elastic fibers in the cytoplasm of multinucleated giant cells (Fig. 5).

Clinical and histopathological findings were consistent with annular elastolytic giant cell granuloma. The patient was initially treated with a medium potency topical corticosteroid in combination with tacrolimus 0.1% ointment, with mild benefit. At 6-month follow-up, the authors performed one treatment of intralesional steroids at the border of the lesions, with some improvement in the erythema and stability of the size of the lesions.

## Discussion

Annular elastolytic giant cell granuloma (AEGCG) is an uncommon granulomatous cutaneous disease that usually affects sun-exposed skin but may also occur in sun-protected areas.^1^ It is associated with an alteration of elastic fibers and elastophagocytosis, resulting in variable clinical presentations.^2^ Both genders are affected but there is a slight female preponderance. It typically manifests as annular plaques with raised erythematous borders and a slight atrophic and hypopigmented center. Non-scarring alopecia has been reported as a possible presentation of AEGCG.^1^

Diagnosis may be difficult due to the similar clinical features to other granulomatous dermatoses, such as sarcoidosis, classic granuloma annulare and necrobiosis lipoidica.^1^ Histopathology is the gold standard for diagnosis.^1^ Diagnosis is based on the histological presence of granulomatous inflammation, absence of necrobiosis, and presence of multinucleated giant cells with more than one area of elastophagocytosis .^2^ Besides the annular form, giant cell elastolytic granuloma can also be classified in papular, reticular or mixed forms, according to the clinical presentation ^2^

Dermoscopy of the lesions may help to narrow down the clinical differential diagnosis. The dermoscopic hallmark of granulomatous skin diseases consists of a structureless yellowish-orange area, distributed in a focal or diffuse pattern, due to the presence of a granulomatous infiltrate in the dermis.^3^ However, dermoscopy findings of this particular disorder are scarce in the literature. In the studied patient, the authors observed an erythematous background with multiple empty follicular ostia with follicular yellowish to orange dots, among the central portion of the patch. No scale was observed. Yellowish/orange dots were previously reported in trichoscopy of scalp sarcoidosis, and the authors associated them with the presence of granulomas in the superficial dermis.^4^ Female pattern hair loss (FPHL) may present with yellow dots but the lack of these findings on the unaffected scalp of the patient, along with the absence of anysotrichosis, make the diagnosis of FPHL unlikely.

This dermoscopy report of AEGCG involving the implantation hair line widens the differential diagnosis of scalp dermatosis with yellowish-orangish dots. The authors believe that it may present a clue to the diagnosis of this entity. More observational reports are needed to confirm the present observations.

## Financial support

None declared.

## Authors’ contributions

Tiago Fernandes Gomes: Data collection, analysis, and interpretation; critical literature review; preparation and writing of the manuscript

José Carlos Cardoso: Approval of the final version of the manuscript; data collection, analysis, and interpretation; Manuscript critical review.

Victoria Guiote: Approval of the final version of the manuscript; data collection, analysis, and interpretation; intellectual participation in propaedeutic and/or therapeutic management of studied cases; manuscript critical review.

## Conflicts of interest

None declared.
